# Treatment of Fetal Arrhythmias

**DOI:** 10.3390/jcm10112510

**Published:** 2021-06-06

**Authors:** Alina Veduta, Anca Maria Panaitescu, Anca Marina Ciobanu, Diana Neculcea, Mihaela Roxana Popescu, Gheorghe Peltecu, Paolo Cavoretto

**Affiliations:** 1 Obstetrics and Gynecology Department, Filantropia Clinical Hospital, 11171 Bucharest, Romania; alina.veduta@gmail.com (A.V.); ciobanu.ancamarina@gmail.com (A.M.C.); dianamarianeculcea@gmail.com (D.N.); gheorghe.peltecu@umfcd.ro (G.P.); 2 Obstetrics and Gynecology Department, Carol Davila University of Medicine and Pharmacy, 020021 Bucharest, Romania; 3 Cardiology Department, Carol Davila University of Medicine and Pharmacy, 020021 Bucharest, Romania; mihaela-roxana.popescu@umfcd.ro; 4 Obstetrics and Gynecology Department, IRCCS San Raffaele Hospital, 20132 Milan, Italy; cavoretto.paolo@hsr.it

**Keywords:** fetal arrhythmia, fetal ultrasound, tachyarrhythmia, bradyarrhythmia

## Abstract

Fetal arrhythmias are mostly benign and transient. However, some of them are associated with structural defects or can cause heart failure, fetal hydrops, and can lead to intrauterine death. The analysis of fetal heart rhythm is based on ultrasound (M-mode and Doppler echocardiography). Irregular rhythm due to atrial ectopic beats is the most common type of fetal arrhythmia and is generally benign. Tachyarrhythmias are diagnosed when the fetal heart rate is persistently above 180 beats per minute (bpm). The most common fetal tachyarrhythmias are paroxysmal supraventricular tachycardia and atrial flutter. Most fetal tachycardias can be terminated or controlled by transplacental or direct administration of anti-arrhythmic drugs. Fetal bradycardia is diagnosed when the fetal heart rate is slower than 110 bpm. Persistent bradycardia outside labor or in the absence of placental pathology is mostly due to atrioventricular (AV) block. Approximately half of fetal heart blocks are in cases with structural heart defects, and AV block in cases with structurally normal heart is often caused by maternal anti-Ro/SSA antibodies. The efficacy of prenatal treatment for fetal AV block is limited. Our review aims to provide a practical guide for the diagnosis and management of common fetal arrythmias, from the joint perspective of the fetal medicine specialist and the cardiologist.

## 1. Introduction

Fetal arrhythmias are detected in 1–2% of pregnancies. Most fetal arrhythmias are benign and transient; however, in some cases, the irregularity of the fetal heart rhythm can indicate a serious condition—either of fetal or maternal origin. Persistent fetal arrhythmia can cause low cardiac output, heart failure, hydrops, and fetal demise [1,2,3,4].

There are three main categories of fetal arrhythmias: (1) an irregular rhythm with a normal fetal heart rate (FHR), as a consequence to premature beats or to conduction anomalies; (2) tachyarrhythmias (defined as FHR > 180 beats per minute-bpm), and (3) bradyarrhythmias (defined as FHR < 110 bpm) [1,2,3,4,5]. Some of the specific terminology used in the article is explained in Appendix A (Table A1).

Premature beats (extrasystoles or ectopic beats) can be atrial, ventricular, or rarely junctional in origin [5].

Premature atrial complexes (PACs; also referred to as premature atrial beats, atrial ectopic beats, or atrial extrasystoles) are caused by early activation of the atrial myocardium because of an impulse generated by an ectopic focus within the atrial myocardium rather than the sinus node. The interval between the last sinus beat and the ectopic beat is shorter than the interval between two normal sinus beats. PACs are triggered from the atrial myocardium in a variety of situations and are almost ubiquitous in the general population [3,6,7].

Premature ventricular complexes (PVCs; also referred to as premature ventricular beats, premature ventricular depolarizations, or ventricular extrasystoles) are triggered from the ventricular myocardium. PVCs are not uncommon and appear in patients without structural heart disease and in those with cardiac disease, independent of severity [3,6,7].

The established range of fetal heart rate is 110–160 bpm. FHR is gestational age dependent, it normally gets slower as the pregnancy advances [8].

Tachyarrhythmias are diagnosed when the fetal heart rate is persistently above 180 bpm and are subdivided into: (1) sinus tachycardia (ST); (2) atrial tachycardia (atrial flutter and atrial ectopic tachycardia); (3) conduction system tachycardia (atrioventricular re-entry tachycardia (AVRT), junctional tachycardia (JT), and atrioventricular nodal re-entry tachycardia (AVNRT); and (4) ventricular tachycardia (VT) [3,6]. This classification can be simplified by dividing tachyarrhythmias into sinus tachycardia (ST), supraventricular tachycardia (SVT), and ventricular tachycardia (VT, rare in fetuses) [3,6,7,9,10,11]. Among fetal SVTs, long ventriculoatrial (VA) interval tachycardia is less frequent than short VA interval tachycardia [12]. The most common fetal tachycardias are paroxysmal supraventricular tachycardia (SVT) either with 1:1 atrioventricular (AV) conduction or atrial flutter (AF) with variable (mostly 2:1) AV conduction [6,7,9,10,11,12,13].

Fetal bradycardia means that the ventricular rate is persistently slower than 110 bpm. This happens frequently in fetal growth restriction, oligohydramnios, or during labor and it is physiological up to a certain extent and duration. However, when the heart rate decreases below 80 bpm for more than 10 min in labor, the risk of neonatal acidemia increases rapidly and significantly [14]. Persistent bradycardia outside labor or in the absence of placental pathology is mainly due to atrioventricular block (AV block) [2,12]. Approximately fifty percent of fetal bradyarrhythmia cases are associated with structural heart defects, and the remaining cases that have normal cardiac structure are often caused by maternal anti-Ro/SSA antibodies [2,12,13,14,15]. The efficacy of treatment for fetuses with AV block is limited compared with that of treatment for fetal tachycardias, particularly when associated with structural defects [12,15].

In utero treatment is necessary when there is significant sustained arrhythmia, and delivery is not an option. Fetal hydrops or foreseeable progression to hydrops is an indication for the treatment of arrhythmias. For most instances, treatment is represented by transplacental medication. The treatment of fetal arrhythmia is a classic example of pharmacological intervention in maternal–fetal medicine. There have been only rare attempts to surgically manage intractable severe fetal arrhythmia [9]. A percutaneously injectable, rechargeable fetal pacemaker was recently produced and successfully tested in an animal model [16].

The aim of this paper is to provide a practical guide to the obstetricians for the diagnosis and management of common fetal arrhythmias.

## 2. The Use of Ultrasound to Assess Fetal Heart Rhythm

Unlike the postnatal setting, where analysis of the heart rhythm is done by electrocardiogram (ECG), the analysis of fetal heart rhythm is based on ultrasound [4]. Despite obvious limitations due to the difficult analysis of the fetal myocardial electric signal, a detailed analysis of fetal arrhythmia is possible using M-mode and Doppler echocardiography to assess mechanical processes [1,4,12,13,14,15,16,17,18,19,20]. In the presence of a suspected or ascertained arrhythmia, the important features to be evaluated are: (1) FHR; (2) rhythm regularity; (3) the relation and time intervals of the atrial and ventricular contractions [13].

**Figure 1:**
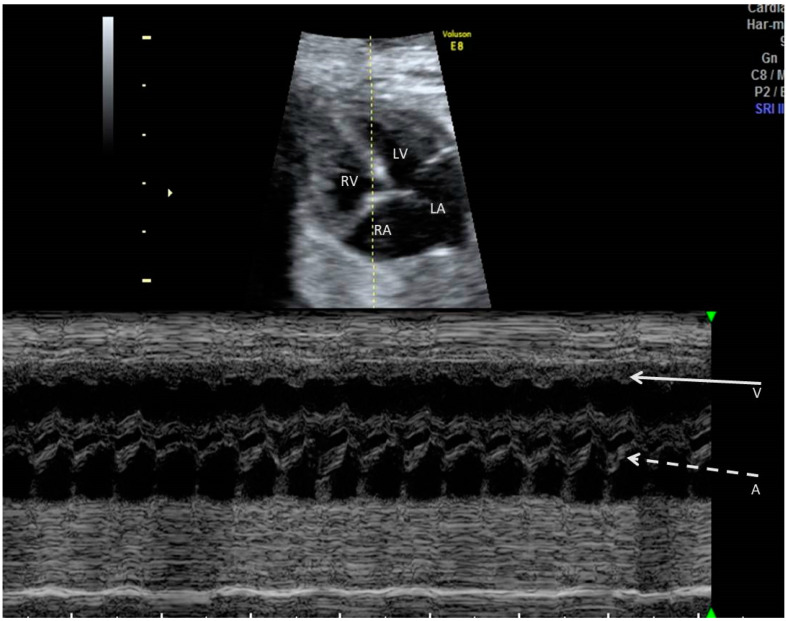
M-mode, linear representation of adjacent cardiac structures motion as a function of time. A 2D relevant image of the fetal heart is first obtained; then, the M-mode cursor is placed at the targeted location on that image. In case of arrhythmia, the M-mode cursor is usually placed across an atrium and a ventricle, so that the relationship of atrial to ventricular contractions is recorded. Arrows indicate ventricular (V) and atrial (A) contractions.

M-mode echocardiography gives a linear representation of adjacent cardiac structures’ motion as a function of time. To obtain a useful M-mode recording, a 2D relevant image of the fetal heart is needed; then, the M-mode sampling line is placed at the targeted location on that image. In case of arrhythmia, the M-mode cursor is usually placed across an atrium and a ventricle, so that the relationship of atrial to ventricular contractions is recorded (Figure 1). While this method has very good temporal resolution, the onset and peak of atrial or ventricular contraction are not well defined on the M-mode, which limits its use in the measurement of the AV time intervals [4]. Furthermore, the application of the M-mode is limited in non-ideal fetal positions with significant shadowing of the cardiac structures [13,18].

**Figure 2:**
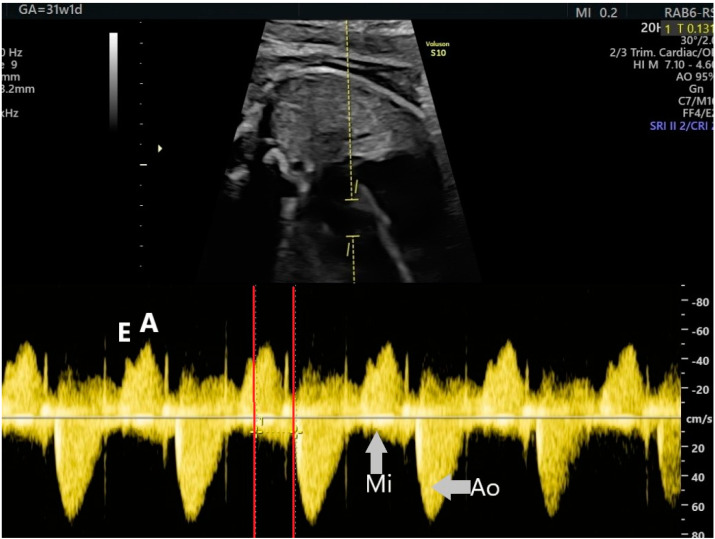
The mechanical PR interval measured with a wide cursor on the mitral and aortic valves. A—the active filling of the ventricles during atrial systole, Ao—aortic flow, E—the passive filling of the atria during generalized diastole, Mi—mitral flow.

Pulsed Doppler echography is the method most used by fetal medicine specialists to assess the AV time intervals, on fetal echocardiography [13,14,15,16,17,18,19,20]. For simultaneous recording of left ventricular inflow and outflow waveforms, a wide cursor (3–4 mm) is placed on the mitral and aortic valves, in the 5-chambers view of the heart. Spectral Doppler waveforms for both the mitral and the aortic valves are recorded during a full cardiac cycle. The trace speed should be increased up to 4–5 cm/s to have a good representation of each waveform. During a normal cardiac cycle, the filling of the ventricles during generalized diastole (E wave), the active filling of ventricles during atrial systole (A wave), and blood ejection into the aorta during ventricular systole will be recorded (Figure 2). The A wave corresponds to the P wave of the ECG. The AV interval, which is the time between atrial and ventricular contraction, can be measured from the onset of the A wave to the onset of the aortic flow (V wave). The AV interval represents a mechanical analog to the electrical PR interval of the ECG; it is often described by fetal medicine specialists as the mechanical PR interval (Figure 2). The mechanical PR interval is normally about 0.12 s (gestational age, sex, and heart rate dependent), with the upper limit of normality of 0.14 s, or 0.15 s in later gestation [9,12,13,18,19,20].

Other rarely used sampling sites for the simultaneous recording of the atrial and ventricular waveforms are: the superior vena cava and aortic artery, by rotating 90° from the 4-chamber view [12,19]; the pulmonary venous and pulmonary arterial Doppler on the 4-chamber view [20].

## 3. Irregular Rhythm with Normal HR

**Figure 3:**
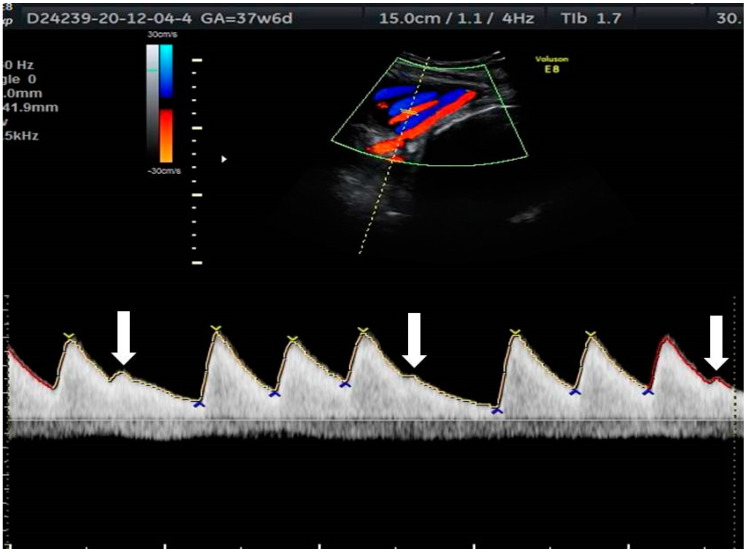
Atrial ectopic beats (indicated by arrows) on the umbilical artery Doppler waveform.

Irregular rhythms due to ectopic beats are the most common type of fetal arrhythmias, most often seen in the third trimester. Premature atrial complexes (PACs) are extra beats in which the ectopic focus originates in the atria, and premature ventricle complexes (PVCs) are those that originate in the ventricles. In utero, the prevalence of PAC to PVC is about 10:1; PACs are difficult to differentiate from PVCs. Premature ventricular beats are not preceded by atrial contractions. PACs may be followed by a ventricular contraction when conducted to the ventricles, or not, in cases of blocked PACs [1]. Ectopic beats are clearly seen on fetal and umbilical artery Doppler waveforms (Figure 3). Nonetheless, tracing of the cardiac flow waves is preferable whenever possible, as umbilical artery Doppler waveforms are less informative on the origin of the ectopic beats.

Isolated ectopic beats are generally benign, regardless of the chamber of origin. Fetal ectopy is associated with congenital cardiac defects in only 1% of cases, and fetal echocardiography is not always recommended. In general, antiarrhythmic therapy is not needed for isolated PACs or PVCs. In most cases, these arrhythmias resolve spontaneously before delivery [1]. However, ectopic beats can cause arrhythmia if there is a substrate for reentry tachycardia [11]. Of note, due to immaturity, aberrant conduction pathways are more often found in the fetal myocardium than in the adult myocardium [21].

Irregular rhythms due to conduction abnormalities (second degree heart block) are more concerning and diagnosis should be followed by immediate referral to a fetal cardiologist.

The distinction between blocked ectopic beats and conduction anomalies can be difficult to make. If the atrial rate is faster than the capacity of the AV node to conduct impulses, some of the impulses are blocked. Atrial tachycardia with functional atrioventricular block often produces a lower than normal ventricular rate (about 70 bpm), which is difficult to differentiate from the second-degree AV block. Bigeminal blocked PACs (blocked atrial bigeminy—BAB) is a particular form of atrial tachycardia with atrioventricular block.

Rarely, the irregular rhythm with normal heart rate can be caused by junctional or ventricular escape beats caused by sinus node dysfunction [11].

## 4. Tachyarrhythmias

Fetal tachycardia is defined as a heart rate (HR) faster than 180 bpm. It is called sustained if the arrhythmia is present for more than 50% of the examination time or intermittent when periods of normal HR and periods of tachycardia alternate [1,3].

Descriptors of atrial tachyarrhythmia, such as “sporadic,” “frequently recurrent,” “non-sustained incessant,” and so on, help characterize the arrhythmia, but may be confusing. These terms describe the frequency and persistence of the arrhythmia, which constitute a continuum of clinical presentations. Usually, the terms ‘intermittent’ as opposed to ‘sustained’ refer to the frequency of the arrhythmia, while the terms ‘paroxysmal’/‘sporadic’ as opposed to ‘persistent’/‘incessant’/‘permanent’ refer to the persistence of the arrhythmia [1].

Pathologically increased fetal heart rate can be encountered in the setting of sinus tachycardia, supraventricular tachyarrhythmia, and ventricular tachycardia [1,6,7,8,9,10,11].

### 4.1. Sinus Tachycardia 

Sinus tachycardia is characterized by a fetal HR > 180 bpm (often < 200 bpm) with normal AV conduction (1:1) [3]. Frequent causes of sinus tachycardia are: maternal thyrotoxicosis, maternal medication (β-agonists), fetal anemia or hypoxia, and infections (chorioamnionitis, fetal cytomegalovirus infection). No specific antiarrhythmic therapy is indicated, but the underlying cause has to be addressed.

### 4.2. Supraventricular Tachyarrhythmia 

*Supraventricular tachyarrhythmia* (SVT) is defined by a non-sinus mechanism of the accelerated heart rate (fetal HR > 180 bpm); this large group of tachycardic disorders includes any non-sinus rapid rhythm that arises from structures above the bundle branches. Supraventricular tachyarrhythmias include atrial flutter and atrial fibrillation [3]. Based on the AV conduction type and time, SVTs are divided into two types:

- Short ‘ventriculoatrial’ tachycardia; it usually involves re-entry mechanisms including atrioventricular re-entry (AVRT) using a bypass tract or atrioventricular nodal re-entry (AVNRT) [2];
- Long ‘ventriculoatrial’ tachycardia—atrial ectopic tachycardia or permanent junctional reciprocating tachycardia (PJRT) [3,12].

**Figure 4:**
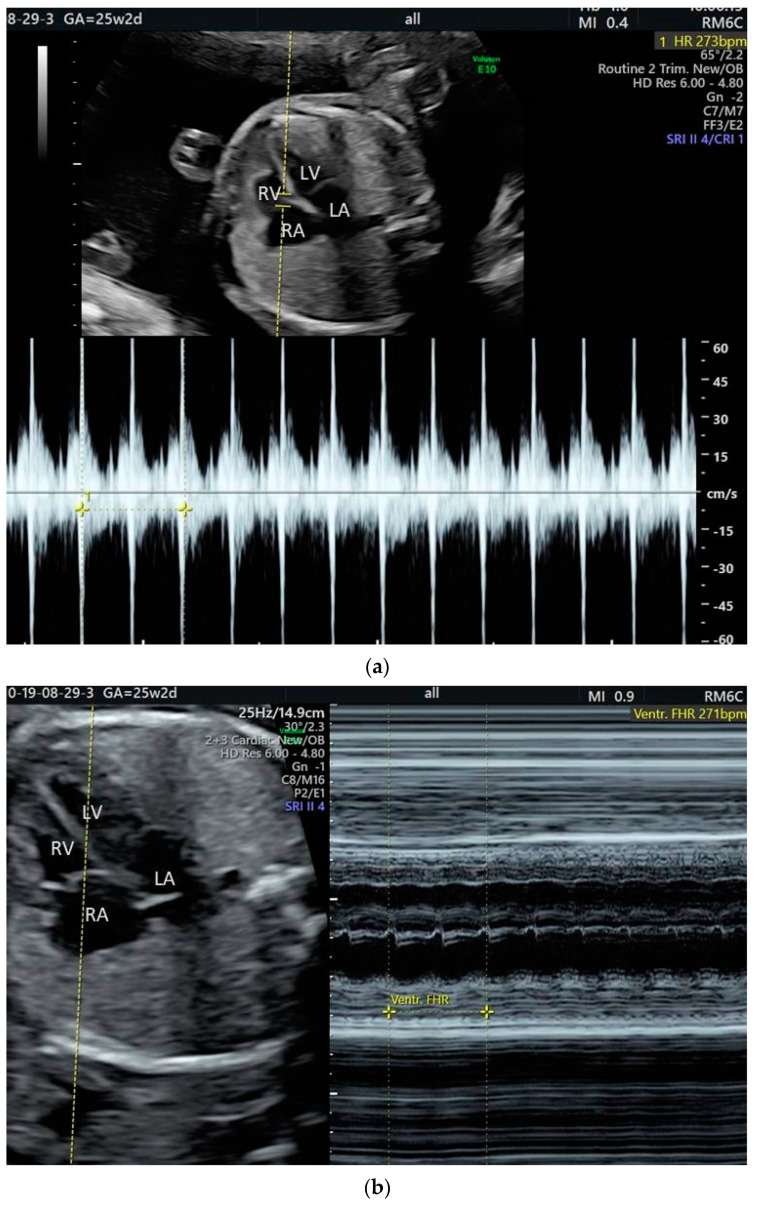
Fetal supraventricular tachycardia: (**a**) Pulsed Doppler analysis; (**b**) M-mode analysis.

The most common fetal tachycardia is re-entry SVT, namely orthodromic atrioventricular re-entry tachycardia (AVRT) (Figure 4a,b) [1,2,3,4,5,6,7]. Of note, the incidence of different forms of SVT is age dependent. Atrioventricular nodal re-entry tachycardia (AVNRT) is very rare in fetuses; in children, its relative prevalence increases with age [21]. AVRT is a paroxysmal tachycardia (PSVT) and it accounts for about two-thirds of fetal tachyarrhythmias [1,10]. This type of tachycardia usually develops between 24 and 32 weeks of gestation [10]. The mechanism is initiated by an ectopic beat [10,11].

**Figure 5:**
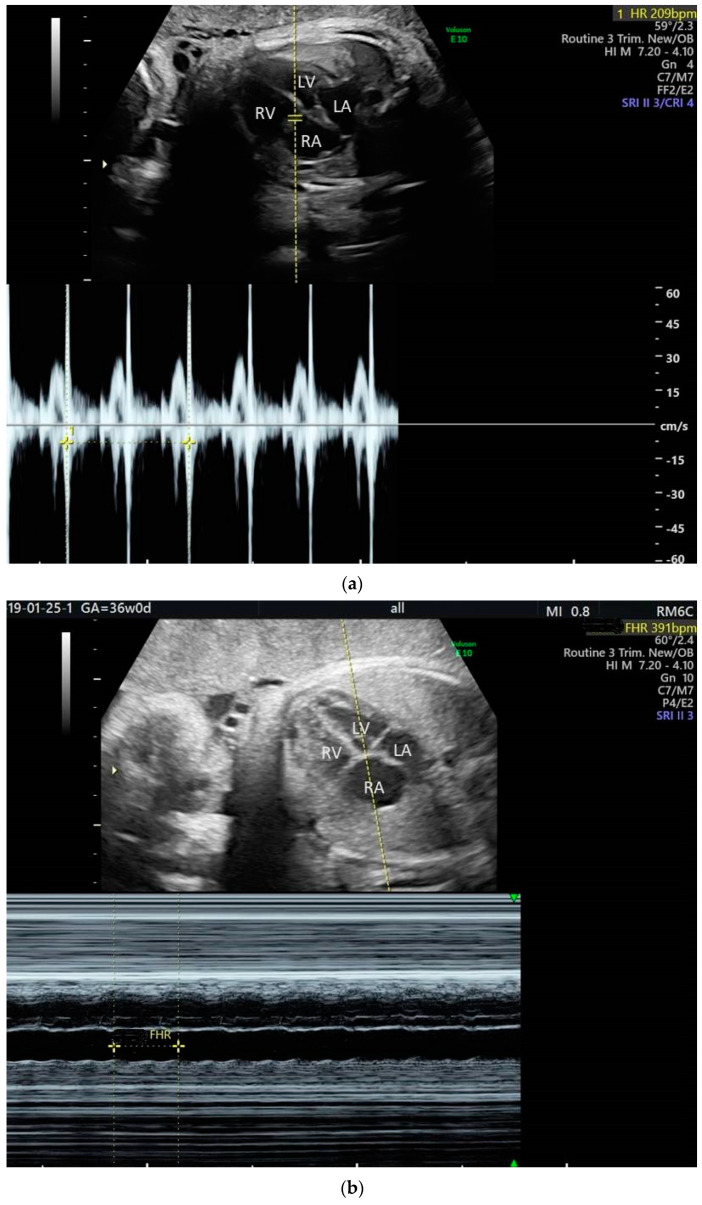
Fetal atrial flutter: (**a**) Pulsed Doppler analysis—ventricular rate; (**b**) M-mode analysis—atrial rate.

Atrial flutter (AF) is the second most common fetal tachycardia (up to 30% of cases) [9,22,23]. It is defined by high atrial rates (300–500 bpm) and slower ventricular response in the setting of variable atrioventricular conduction (Figure 5a,b) [2]. Usually, AF is diagnosed later in gestation [1,3,20]. AF can be associated with myocarditis, anti-Ro (SSA) antibodies, and structural heart disease; fetuses with AF develop hydrops in about 15% of the cases [1,22,23,24]. Postnatal data shows that up to 20% of individuals with AF also have other types of SVT [25,26].

The treatment of SVT consists of antiarrhythmic medication; the substances used to treat fetal SVT are digoxin, flecainide, sotalol, and, more rarely, amiodarone. Correcting abnormal maternal electrolytes levels and vitamin D deficiency can help the conversion of tachycardia and maintain the sinus rhythm of the fetus [10].

Three options are available when tachyarrhythmias are diagnosed in a fetus: delivery, in utero monitoring, or in utero treatment [1,3,9,27].

The decision to treat fetal SVT depends on several factors such as the mechanism and persistence of tachycardia, gestational age, and associated congenital heart disease (CHD) [1,25]. Practically, the probability of progression to hydrops is the most important factor to consider when deciding the management of prenatally diagnosed arrhythmia. Although it is difficult to predict if a fetus will develop hydrops and when it will occur, several parameters are useful in assessing the risk of progression to hydrops. Early gestational age, high ventricular rates, and incessant tachycardia are factors determining hydrops progression [1,28].

Delivery is a valid option for fetuses at term and near term. Postnatal treatment of tachyarrhythmia is usually effective. There is little reason to pursue transplacental drug therapy for the term fetus with tachyarrhythmia but no hydrops. Some authors advocate that hydrops in a near-term fetus is a clear indication for emergent delivery [3]. Nonetheless, consideration should be given to the fact that in utero conversion of arrhythmia often allows for hydrops remission. Therefore, a short trial of an effective agent (flecainide or sotalol rather than digoxin) may be considered rather than delivery, for the hydropic fetus at term. If the attempt to slow the FHR is ineffective, delivery in 48 to 72 h is indicated. Hydrops that does not respond to treatment represents an indication for delivery, for term as well as for premature fetuses. Any type of fetal hydrops can cause the rare maternal mirror syndrome. Severe mirror syndrome or severe preeclampsia indicates delivery at any gestational age.

Tachyarrhythmia is sometimes well tolerated, particularly the intermittent type which manifest near term with moderate ventricular rates (up to 220 bpm). In cases with low risk of progression to hydrops, close monitoring without transplacental therapy can be considered [1,3]. In-hospital monitoring of the fetus for the initial 12/24 h to evaluate fetal well-being and frequency of the arrhythmia is recommended. Once spontaneously intermittent tachyarrhythmia has been documented in a fetus without structural heart disease, outpatient monitoring by repeated ultrasound once or twice a week is possible [3]. Hydrops and neurologic sequelae have rarely been reported in case of intermittent fetal tachycardia [3,23,27,28,29,30].

In utero therapy is the recommended management for fetuses with hydrops or at high risk of developing hydrops (sustained tachycardia with ventricular rates more than 220 bpm). The goal of therapy is not necessarily to stop the SVT, but to slow the ventricular rate enough to achieve a good cardiac output. As a rule, the motivation for in utero treatment increases with prematurity. In very preterm fetuses (less than 32 weeks of gestation), combination pharmacological therapy in higher than usual doses or direct fetal treatment with amiodarone, adenosine, or digoxin should be considered instead of delivery if the initial course of transplacental therapy fails. As mentioned before, there is also good reason for a short trial of an effective agent, in cases of hydropic fetuses at or near term.

The practice of fetal medicine centers varies widely in respect to the drug chosen as initial treatment for fetal SVT. All of the following drugs: digoxin, flecainide, sotalol, and amiodarone have been used as first-line transplacental therapy in SVT [1,9,10,12,13,31,32,33,34,35,36,37,38,39,40,41,42,43,44,45,46,47,48]. The effectiveness of transplacental treatment is dependent on pharmacokinetics, its capacity to cross the placenta, and fetal bioavailability [1,5]. Importantly, in fetuses with hydrops, the transplacental passage of digoxin is slow. The mechanism of tachycardia is also important for the choice of treatment; it seems that for fetal PSVT with long VA interval, digoxin is rarely effective [12].

For a longtime, digoxin has been used as first line therapy for fetal SVT, but none of the recent studies and meta-analyses supports this idea [31,32,33,34,35,36,37,38,39,40,41,42,43,44,45]. Nonetheless, digoxin has some practical advantages if treatment is considered for moderate fetal disease [1,3]. In resource rich settings especially, there is expertise to initiate digoxin without continuous cardiac monitoring and serum digoxin levels are considered easy to monitor. In contrast, continuous inpatient cardiac monitoring is the standard practice for medical cardioversion with flecainide or sotalol. Therefore, continuous inpatient maternal monitoring is considered good practice for initiation of transplacental therapy with flecainide or sotalol. If fewer resources are available, treatment with the β-blocker sotalol can actually be easier to monitor [39].

According to systematic data, both flecainide and sotalol are more effective than digoxin in converting fetal SVT, and even more so in hydropic fetuses. A study from 2016 showed the superiority of flecainide over digoxin, especially in cases of long VA tachyarrhythmia [41]. An important study by Jaeggi et al. showed that flecainide was the most efficient first-line medication for fetal SVT, overall [42]. Two relatively recent meta-analyses showed that flecainide and sotalol achieve better results than digoxin in treating fetal SVT. A meta-analysis of ten studies concluded that flecainide has a higher rate of SVT termination than digoxin and both flecainide and sotalol are better than digoxin in fetuses with hydrops and tachyarrhythmia [43]. In a meta-analysis of 21 studies on the transplacental treatment of fetal tachycardia, both flecainide and sotalol were more effective than digoxin for conversion of any fetal tachycardia to sinus rhythm, and the difference was greatest in hydropic fetuses [44]. The experience of a large center with very good results in fetal SVT treatment shows that drug change or multidrug therapy were necessary in 68% of cases where digoxin was used as first-line therapy [45]. In the particular setting of atrial flutter, treatment with flecainide or sotalol aims to terminate the arrhythmia, while treatment with digoxin aims to slow the ventricular rate. Studies suggest that sotalol might be the drug of choice to terminate or control fetal AF [42]. However, we have to take into account that available data regarding fetal AF treatment is not from randomized trials but rather from case series. In all instances, personal experience is an important factor in the management of fetal SVTs and patients would be best served by the input of a complex team of specialists in fetal medicine, obstetrics, neonatology, and pediatric and adult cardiology.

The loading dose for digoxin is 1–2 mg, divided over 24 h, followed by the measurement of the digoxin serum level. The target serum level for digoxin in general cardiology is 1 to 2 ng/mL; for the transplacental treatment of fetuses, a maternal serum level in the higher range, around 2 ng/mL, seems advisable. Maintenance doses usually need to be higher in pregnant versus in nonpregnant women, ranging from 0.5 to 0.75 mg daily given in three daily doses, because of increases in blood volume and in the renal clearance. The maintenance dose is determined by titrating to the fetal response, over several days.

The usual initial dose for flecainide is 250 mg per day, orally in three doses (100, 50, 100 mg). It can be increased to 300 mg daily if needed, under strict continuous maternal surveillance.

The usual initial dose for sotalol is 240 mg per day, orally in three equal doses. It can be increased if needed, under strict continuous maternal surveillance.

An amiodarone loading of 12 to 13 g over a week has been reported to achieve conversion to sinus rhythm, after a mean interval of six days. Amiodarone is very rarely used as first-line therapy, as it has a long half-life and can cause neonatal hypothyroidism [47,48].

There is little data to document the duration of transplacental therapy, until cardioversion. Available data shows that medical cardioversion of fetal SVT is achieved within 48 h, in at least 50% of cases, irrespectively of the drug used [46]. The data on the actual mean time to cardioversion is sparse and sometimes contradictory. One study reported that the median time to conversion of SVT was 3 days with digoxin, 4 days with flecainide, and 12 days with sotalol [42]. Another study reported a median of one day for cardioversion with sotalol [39]. As amiodarone crosses the placenta slowly, it takes a relatively long time of amiodarone treatment to achieve the therapy goal. The time to conversion is significantly longer in AF cases and in fetuses with hydrops [42]. The practical issue related to this subject is how long to continue a treatment regimen if no improvement (conversion to sinus rhythm or reduction of the ventricular rate) is seen. In general, the more unstable the fetus is, the more likely one is to quickly (after 72 h) abandon a treatment that seems inefficient.

If the first-line therapy is inefficient, another drug can be used or added to the first-line drug. Combination therapy usually consists of digoxin and one of the other drugs. Flecainide increases digoxin serum levels, thus digoxin dose adjustment is needed when adding flecainide to digoxin. Direct fetal administration (intramuscular digoxin or intravenous amiodarone by cordocentesis) is rarely considered, as most SVTs are eventually controlled with transplacental medication.

Successful treatment (control of the SVT) is defined by either conversion to sinus rhythm or reduction of the ventricular rate by more than 15% [41].

Once the arrhythmia is controlled, outpatient monitoring is possible, and medication can be given in two rather than three daily doses. Attempts to taper the medication after a long time of sinus rhythm or before delivery are sometimes made; this is justified in the case of sotalol, which can have significant neonatal adverse effects.

If the conversion to sinus rhythm is achieved, there are no specific recommendations for the delivery and the child can be evaluated after birth by the pediatric cardiologist, on an outpatient basis. If sustained tachycardia is present during delivery, experienced support personnel from neonatal intensive care and cardiology should be available at the time of birth. Therefore, in utero transfer to a comprehensive center is always recommended, under the circumstances.

There is increasing awareness of the potential risks of proarrhythmia to both the mother and the fetus [49]. Before treatment initiation, assessment of the mother by a cardiologist is warranted. Initiation of the treatment is usually done in hospital, sometimes with continuous cardiac monitoring for 48 h. After initiation, monitoring consists in daily electrocardiograms. Daily electrocardiograms are obtained to look for PR prolongation (suggests digoxin toxicity), QT prolongation (suggests flecainide or sotalol toxicity), or QRS prolongation (suggests flecainide or sotalol toxicity) [49].

Development of preeclampsia with maternal renal impairment is, in most instances, an indication to stop the administration of any renally-cleared proarrhythmogenic substances.

### 4.3. Ventricular Tachycardia 

Ventricular tachycardia (VT) is very rare in fetuses. It has been documented in association with myocarditis, cardiac tumors, ventricular aneurysms, long QT syndrome, and unstable atrioventricular block [1,10,50,51,52]. There is complete dissociation of atrial and ventricular contraction, and the atrial rate is slower than the ventricular rate. Expectant management is an option for stable fetuses. First-line therapy of VT consists of maternal intravenous magnesium sulphate [9,53]. Transplacental treatment with lidocaine, dexamethasone, beta-blockers (propranolol), flecainide, and amiodarone has been described [10,54]. One recent paper reported that, in the absence of tumors or cardiomyopathy, beta blockers (excluding sotalol) should be the first-line therapy for fetal ventricular tachycardia [55].

Other substances, such as verapamil (no longer prescribed for infants) or procainamide, have been sometimes used to treat fetal tachyarrhythmias.

The usual doses and regimens as well as the adverse reactions of some of the agents used to treat fetal tachyarrhythmia are presented in Table 1 [54].

## 5. Bradyarrhythmias

The obstetric definition of fetal bradycardia is sustained FHR of less than 110 bpm for more than 10 min [2,10]. FHR is dependent of gestational age, and it decreases significantly as gestation progresses from an average of 141 bpm at 32 weeks of gestation to 137 bpm at 37 weeks of gestation [2,8,10]. Persistent low heart rate may be normal or may be a marker for significant conduction disease [6,9,10,54]. A persistent ventricular rate of less than 60 bpm is usually associated with complete heart block, while rates between 60 and 90 bpm can be due to non-conducted bigeminy or second-degree block.

Persistent low fetal heart rate can be caused by sinus, low atrial or junctional bradycardia, blocked ectopic beats, long QT syndrome, or atrioventricular block (second or third degree).

### 5.1. Sinus Bradycardia 

Sinus bradycardia is rare [2,3]. It is defined by 1:1 atrioventricular conduction with a slow atrial rate [2,3].

It can be caused by structural heart disease in heterotaxy, long QT syndrome (LQTS), or noncardiac conditions such as maternal medication, maternal hypotension, hypoglycemia and hypothermia, maternal conditions including fetal–maternal infections, and pregnancy or delivery complications. The causes of sinus bradycardia are presented in Table 2. It is important to recognize the causes of sinus bradycardia in order to avoid emergent delivery because of presumed fetal distress [2,10]. Fetal therapy is not necessary for sinus or low atrial bradycardia, but fetal well-being should be attentively assessed and monitored. The nonstress test is likely to be nonreactive with sustained sinus bradycardia, but variability may be normal.

### 5.2. Long QT Syndrome

Congenital long QT syndrome (LQTS) is a disorder of ventricular myocardial repolarization caused by genetic ion channelopathies or by an imbalance in the sympathetic innervation of the heart [56,57,58,59]. LQTS should be suspected in unexplained fetal demise after 20 weeks of gestation or stillbirth, particularly in cases with positive family history [10,56]. The most common presentation of LQTS in fetuses is bradycardia, but LQTS can also present with second degree block, alternating tachycardia and bradycardia, torsades de pointes, or ventricular tachycardia [9,10,53,54]. ECG of the parents is recommended if LQTS is suspected in a fetus. Prenatal diagnosis is possible in families with known mutations. If the syndrome is confirmed in the fetus or in the mother, close observation is recommended and drugs that lengthen the QT interval are contraindicated [10]. Electrolyte and vitamin D deficiency should be corrected [10]. Therapy is not necessary for bradycardia; magnesium sulphate, mexiletine, lidocaine, or betablockers can be tried for torsades de pointes arrhythmia [57].

### 5.3. Blocked Ectopic Beats

Ectopy causing irregular fetal heart rhythm has been discussed above. Blocked ectopic atrial beats can lead to sustained bradycardia, especially in the case of bigeminy or trigeminy. It is very important to distinguish blocked ectopic beats from complete heart block. In complete heart block, the atrial rhythm is regular, and in atrial ectopy with blocked beats, the atrial rhythm is irregular. Abrupt shifts from low heart rates to approximately 120 to 160 bpm suggest blocked premature contractions during the bradycardic periods. Reasonably close monitoring (weekly or every two weeks) should be provided by the fetal medicine specialist as long as the atrial ectopy persists.

### 5.4. Heart Block

Atrioventricular block (AV block) refers to altered (delayed, intermittent, or absent) conduction between the atria and the ventricles [2,10]. The atrial rate is regular and normal, but the ventricular rate is slow because of the AV dissociation [2,10,51]. There are three degrees of block: first, second, and third (complete) [2,6,10]. Congenital heart block (CHB) is rather rare and it is usually associated with fetal structural heart disease or maternal autoimmune disease [2,12,60].

First-degree AV block is not truly a block, but a delay of the conduction between the atria and the ventricles, seen as prolonged PR interval. As there is no interruption of the conduction between the atria and the ventricles, it might be more appropriate to use the term ‘prolonged AV conduction’ rather than ‘AV block’, to describe this pathology. In fetuses, the mechanical PR interval can be measured, as described above. When measured at the mitral/aortic valves, the normal values for the fetal PR are 0.12 ± 0.02 s [9], with an upper limit (cutoff) of 0.14 s, or 0.15 s if the 99th centile of the normal range at advanced gestation is considered. Measurement of the PR interval is not routinely done during fetal morphology assessment or during fetal echocardiography either; therefore, fetal first-degree AV block is rarely diagnosed in the general population. In mothers with conditions predisposing to fetal heart block, it is recommended to measure the PR interval, between 18 to 28 weeks of gestation, so that damage to the cardiac conduction system can be recognized early, monitored, and managed in utero, if considered necessary [61].

**Figure 6:**
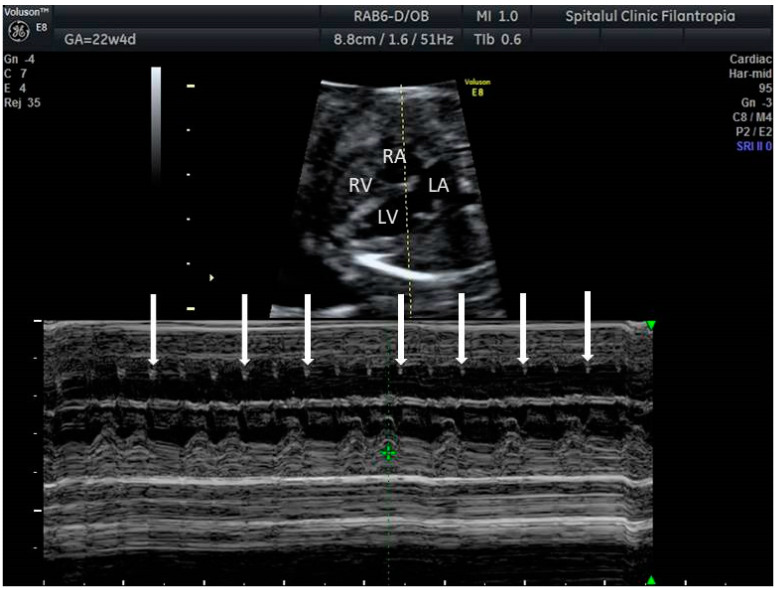
Second degree fetal heart block. Arrows indicate atrial contractions.

Second-degree heart block is uncommonly diagnosed in the fetus, as it represents a transient phase in the deterioration of the fetal cardiac conduction system. Second-degree AV block in the fetus often presents with lengthening of the mechanical PR interval from one beat to the next—a Mobitz I type of second-degree block (Figure 6), which, in theory, has a lower risk of progressing to complete atrioventricular block (CAVB). Nonetheless, fetal second-degree AV blocks very often progress to CAVB. Referral to cardiology is warranted. When second-degree fetal AV block is diagnosed incidentally, the mother should be tested for anti-Ro/SSA and anti-La/SSB antibodies.

**Figure 7:**
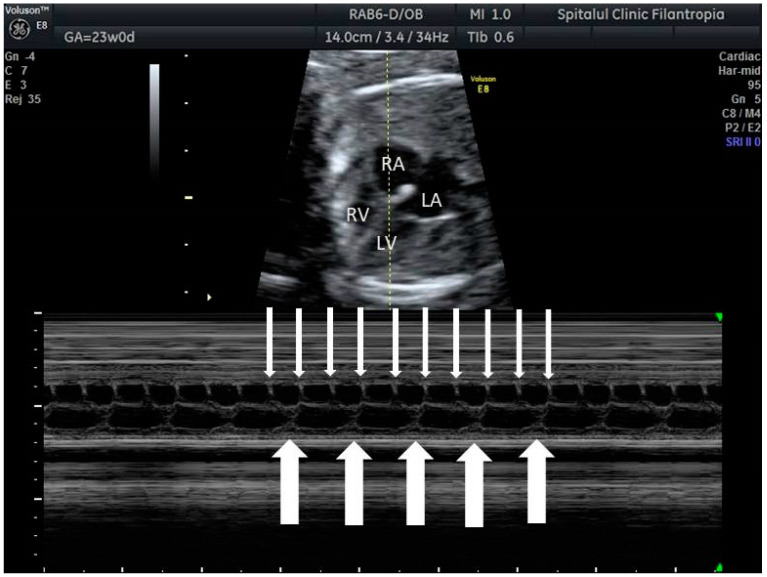
Complete (third degree) fetal heart block. Thin arrows indicate atrial contractions, thick arrows indicate ventricular contractions; there is AV dissociation.

Complete atrioventricular block (CAVB) is an important cause of sustained fetal bradycardia [2,10,12]. In CAVB, there is a complete dissociation between the atria and the ventricles because of the absence of atrioventricular conduction (Figure 7); ventricular rate is typically 50–80 bpm, but it can be lower. Even at typical ventricular rates and very often at rates less than 50 bpm, cardiac dysfunction and fetal hydrops are noted.

About 50% of fetuses with CAVB have complex congenital heart disease (CHD). Heterotaxy (left isomerism) and cardiac malformations with L-looped ventricles (L-transposition of great arteries) are the main types of CHD associated with CAVB [2,10,12]. Left atrial isomerism and transposition can be sometimes recognized on ultrasound from the first trimester of pregnancy [62,63,64]; yet, the unpredictable evolution to CAVB cannot be prevented in such cases. Fetal and neonatal mortality is high in CAVB associated with cardiac malformations, particularly in the setting of hydrops [60].

CAVB in structurally normal hearts is mostly due to immune-mediated inflammation and fibrosis of the conduction system. The most frequent antibodies responsible for CAVB in the fetus are maternal SSA/Ro and/or SSB/La antibodies, which can cross the placenta [15,65]. Autoimmune CAVB usually develops between 18 and 28 weeks of gestation, the highest risk period being between 18 and 24 weeks of gestation [10,15].

Isolated non-autoimmune fetal complete atrioventricular block is rare and usually familial. It seems to be associated with a better survival rate than the autoimmune one [66].

Third-degree block represents about 80% of cases of CHB, and the rest is evenly distributed between first- and second-degree block.

Treatment of AV block depends on the etiology, presence and stage of heart failure, and ventricular rate [6,10]. There are few options for prenatal therapy. In particular, congenital heart block associated with hydrops fetalis is a life-threatening condition with no effective treatment options. Preterm delivery at the onset of hydrops can be considered, but pacing very small premature infants is difficult and frequently unsuccessful [2,60].

In utero therapy options for fetal heart block include: beta-sympathomimetics; intravenous immunoglobulin and apheresis; and fluorinated glucocorticoids [2,67,68]. Hydroxychloroquine is used to prevent fetal heart block and neonatal lupus in women with anti-Ro/SSA or anti-La/SSB antibodies with a previous history of fetal heart block or neonatal lupus [12,14]. One study has shown a higher incidence of fetal AV block in winter months, implying that vitamin D maternal levels should be assessed, and deficiencies corrected [69].

Beta sympathomimetics to increase heart rate and stroke volume are often used if the fetal heart rate is less than 50–60 bpm, but no studies demonstrated improved survival rates [2,70].

Immunoglobulins and maternal plasmapheresis have been used, with variable success, to treat autoimmune fetal heart block [2,71,72,73].

The most studied medication for autoimmune fetal heart block is represented by fluorinated glucocorticoids, which cross the placenta (oral dexamethasone 4 to 8 mg per day or betamethasone 3 mg per day). Clinicians use glucocorticoids for various indications, such as second degree AV block, recent onset AV block, or severe cardiac dysfunction and hydrops (extranodal immune-mediated fetal heart disease) [2,73,74,75], but the efficiency of treatment in any of the above circumstances is not proven and questionable.

In a recent meta-analysis [76], fluorinated glucocorticoids did not improve fetal or neonatal survival, while prolonged administration of fluorinated glucocorticoids was associated with fetal and maternal complications. However, this meta-analysis is difficult to interpret. It analyzed all degrees of heart block together, although the natural history and the prognostic significance of each type of block are very different. Complete heart block, once identified, is irreversible despite all therapies attempted to date [2,77]. Second-degree heart block may be reversible, but it also may progress to complete heart block despite therapy. Progression from first-degree block to more advanced heart block in the fetus is difficult to document and seems unpredictable [77]. We discuss the use of glucocorticoids for each type of heart block separately.

There would be equipoise on the recommendation to start transplacental steroids after a diagnosis of first-degree block. One approach is to start steroids for persistent first-degree block [78]. The treatment is discontinued if the first-degree block progresses to complete block and if there is no evidence of extra-nodal disease. If the block remains stable or reverts to normal sinus rhythm, the benefits and the risks of continuing steroid treatment should be carefully weighed. Depending on the fetal echocardiography evolution, dexamethasone may be continued up to 26–28 weeks of gestation, when the critical period reaches the end [78].

Second-degree fetal block is known for its tendency to progress to complete heart block; thus, in utero glucocorticoid therapy is indicated by most experts (intravenous immunoglobulin is an alternative treatment). However, second-degree block can revert to sinus rhythm without treatment, and not all cases respond to treatment. Case series document that both treated and untreated patients can progress, stabilize, or revert to normal conduction. A 2018 systematic review and meta-analysis of five observational studies that included 71 fetuses with second-degree immune-mediated congenital heart block concluded that the use of fluorinated glucocorticoids should not be discouraged, “until more robust evidence is available” [79].

Established third-degree fetal block is an irreversible condition caused by fibrosis and calcification of the AV node. Management of the fetus with CAVB is primarily expectant since the reversal of the established third-degree block has not been documented [2,77]. However, the transition from a normal rhythm to emergent complete CHB and from emergent CHB to established CHB can happen in less than 24 h, and the treatment of the former can revert the conduction to normal [80]. Therefore, in selected cases, daily FHR monitoring can be crucial to diagnose and effectively treat emerging complete CHB. One recent study proved that home monitoring is useful in detecting the emergence of CHB [81]. Otherwise, there is no reason to use fluorinated steroids to prevent disease progression or death in cases presenting with complete isolated heart block caused by anti-Ro/SSA antibodies. If fetal heart injury extends beyond the AV node, on the other hand (endocardial fibroelastosis or global cardiomyopathy), transplacental glucocorticoids might be of benefit and improve survival [73].

There are attempts to develop in utero pacemakers [16,68,82].

Prevention of fetal heart block recurrence in subsequent pregnancy is possible. The efficacy of hydroxychloroquine to prevent recurrent congenital heart block in fetuses of anti-Ro/SSA-positive mothers has been shown by retrospective and prospective clinical studies [83,84]. Hydroxychloroquine is an antimalarial drug that inhibits the ligation of endosomal Toll-like receptors (TLRs) and it is often used in patients with systemic lupus erythematosus. Its preemptive action, in situations others than those of mothers with anti-Ro/SSA antibodies and history of cardiac neonatal lupus is unknown. Yet, many clinicians are prescribing hydroxychloroquine during the first pregnancy in asymptomatic mothers with anti-Ro/SSA antibodies or anti-La/SSB antibodies, as it has minimal side effects for the mother [15].

Delivery of any fetus with heart block and significant bradycardia should take place at a center where support personnel from neonatal intensive care and pediatric cardiology, including a specialist with experience in newborn pacing, are available at the time of birth.

## 6. Discussion

### 6.1. The Fetal Medicine Specialist Point of View

When treating fetal arrhythmias, both the fetus and the mother undergo medical intervention. Transplacental treatment of fetal arrhythmias involves giving medication—in many instances, antiarrhythmic and proarrhythmogenic substances—to the mother, so that the medication can reach the fetus through the placenta. For efficient management, fetal arrhythmias have to be placed in the maternal context, in several respects. Many reversible maternal conditions (infection, hyperpyrexia, thyroid disease, medication, and so on) can cause abnormal fetal heart rhythm. Other maternal conditions (autoimmune disease, long QT syndrome) can be sometimes unveiled by fetal arrhythmia. Side effects of the antiarrhythmic medication have to be tolerated by the mother for the benefit of the fetus.

We think that it is up to the fetal medicine specialist to integrate the maternal and fetal care, under the circumstances. The specialist should set the short-term and the long-term targets for fetal therapy and should be aware of pregnancy-related modifications (particularly of renal function) affecting pharmacokinetics of antiarrhythmics or potential maternal side-effects of medications. The doctor also has to consider that extended medical care for a fetus with uncertain prognosis produces significant parental anxiety, often requiring provision of psychological support. The fetal medicine specialist advises in utero fetal therapy (as opposed to monitoring or delivery), mainly based on the risk of hydrops and intrauterine death, but also taking into account concomitant maternal conditions or the development of pregnancy-related conditions such as preeclampsia.

### 6.2. The Cardiologist Point of View

The role of the cardiologist consists in evaluating the risk/benefit balance from the point of view of the maternal side effects, indicating the medication regimen and monitoring the fetal and maternal cardiac effects of the treatment.

Different specialists use different terminologies, different types of investigations, but they must reach the same conclusion and manage the pregnancy for the benefit of both mother and child. There are differences between the terminology used by a fetal specialist and a cardiologist. The fetal specialist explores the heart through ultrasound and characterizes it using the ventriculoatrial interval, while the cardiologist is used to ECG and its specifics.

Because of this lack of a common point of view, some discrepancies in the current practices of fetal specialists and cardiologists arise. For example, sotalol is not the first option for maternal SVT, but it is the drug currently used for fetal tachyarrhythmias. Currently, there are no common guidelines including both maternal and fetal pathologies. The most recent ESC (European Society of Cardiology) Guidelines recommend the assembly of a pregnancy heart team for better management of cardiovascular diseases during pregnancy [85]. A cardiologist, an obstetrician, and an anesthetist are the indispensable specialists for such a team. Similar teams should be constituted in case of high-risk fetal arrhythmias, with the addition of a pediatric cardiologist, fetal medicine specialist, neonatologist, etc. These teams would be essential for establishing the indications for treatment, balancing the maternal side effects with the benefit for the child, conduct the perinatal care and follow-up, and adjudicate on the prognosis of both mother and fetus [86,87,88].

In conclusion, ultrasound can characterize fetal heart rhythm in detail. For tachyarrhythmias, the type of arrhythmia has less prognostic value than the gestational age at diagnosis or the presence of fetal hydrops. Higher degree heart block, in fetuses with both normal and abnormal hearts, remains difficult to treat.

## Figures and Tables

**Table 1:** Agents used to treat fetal tachyarrhythmia [54].

Condition and Drug	Dose	Adverse Reactions
Supraventricular tachycardia (including atrial flutter)		
Digoxin	LD 1–2 mg per 24 h PO in 3 divided doses MD 375 (500)–750 μg daily POin three equal daily doses	Nausea, severe sinus bradycardia or atrioventricular block, maternal and fetal proarrhythmia
Sotalol	160–480 mg daily in three equal doses PO	Nausea, dizziness, fatigue, bundle branch block, QT prolongation, QRS prolongation, maternal and/or fetal proarrhythmia, neonatal hypoglycemia
Flecainide	250–300 mg daily in three equal doses PO	Visual and central nervous system symptoms, bundle branch block, QT prolongation, QRS prolongation, fetal or neonatal proarrhythmia
Amiodarone	LD 1800–2400 mg daily in equal doses given every 6 h for 48 h; MD 200–600 mg daily PO	Nausea, maternal and/or fetal thyroid dysfunction, photosensitivity, thrombocytopenia, bundle branch block, proarrhythmia, torsades de pointes in fetuses with long QT syndrome
Procainamide	LD 500–600 mg over 20 min iv; MD 2–6 mg/min iv Initially 1250 mg, followed in 1 h by 750 mg, then 250–1000 mg every 3–6 h PO	Nausea, hypotension, proarrhythmia, platelets abnormalities
Ventricular tachycardia		
Magnesium sulfate	LD 2–4 g iv followed by 1–2 g/h iv	Fatigue, central nervous system symptoms, stop for loss of patellar reflex at levels of 3.5–5.0 mmol/L cardiac arrhythmias at high levels
Lidocaine	LD 1.0–1.5 mg/kg iv followed by 1–3 mg/min iv	Central nervous system symptoms
Propranolol	40–80 mg every 8 h PO	Fatigue, bradycardia, hypotension
Dexamethasone	LD 4–8 mg PO; MD 4 mg daily PO	Maternal effects similar to other corticosteroids, possible negative influence on the development of the fetal brain

LD—loading dose; MD—maintenance dose.

**Table 2:** Causes of sinus bradycardia.

Sinus node dysfunction in left atrial isomerism
Congenital LQTS
Sinus node inflammation or fibrosis in viral myocarditis or autoimmune collagen diseases (SSA/Ro [+] or SSA/Ro and SSB/La [+] antibodies)
Fetal distress
Fetal hypoxia
Fetal acidosis
Maternal hypotension
Maternal hypoglycemia
Maternal hypothermia and cardiopulmonary bypass
Maternal treatment with beta blockers or sedatives

## Appendix A

**Table A1:** Cardiology terminology explained for the fetal medicine specialist [86,87,88].

escape atrial beats	heart beat originating in an ectopic focus in the atrial myocardium, after a long sinus pause; the sinus pause can be caused by sinus node exit block, sinus node arrest and so on; the escape rhythm is slower than the sinus rhythm
premature atrial complexes/beats (PACs)	early activation of the atrial myocardium by an impulse coming from an ectopic focus within the atrial myocardium, not from the sinus node; the interval between the last sinus beat and the ectopic beat is shorter than the interval between two sinus beats
atrial bigeminy/trigeminy	a rhythm in which every other beat (bigeminy) or every third beat (trigeminy) is an ectopic beat (PAC)
supraventricular tachycardia (SVT)	an abnormally fast heart rhythm originating above the ventricles; in a broader sense, it includes atrial flutter and atrial fibrillation
ectopic atrial tachycardia	sometimes referred to as atrial tachycardia or primary atrial tachycardia, it is a rhythm generated by an ectopic atrial focus, which is faster than the normal heart rate (180 bpm in fetuses)
reentry tachycardia	tachyarrhythmia caused by the repetitive propagation of an excitatory wave through a circular path, returning to its site of origin to reactivate that site
reciprocating tachycardia	reentry tachycardia
paroxysmal tachycardia	also called sporadic, it is a self-limited reentry tachycardias initated by ectopic beats and terminated by functional block
paroxysmal SVT	also called sporadic, those intermittent SVTs with abrupt onset and offset and a regular ventricular response, mostly due to reentry, eg AVRT
incessant tachycardia	nonparoxysmal tachycardia, most often caused by enhanced/abnormal automaticity or triggered activity
sustained tachycardia	arrhythmia present for more than 50% of the examination time
intermittent tachycardia	arrhythmia present for less than 50% of the examination time
orthodromic tachycardia	SVT in which the reentrant circuit conducts impulses down (antegradely) through the AV node and the His–Purkinje system to the ventricles, and then up (retrogradely) through the accessory pathway (narrow complex tachycardia)
antidromic tachycardia	rare SVT in which the reentrant circuit conducts impulses down (antegradely) through the accesory pathway to the ventricles, and then up (retrogradely) through the AV node (wide complex tachycardia)
short VA tachycardia	If the RP interval is less than one-half of the RR interval, the tachycardia is considered a short RP (short VA) tachycardia. If the P wave is retrograde, AV reentry with the common slow—fast conduction type is most probable. The slow—fast pattern gives rise to retrograde P waves that is very close or even masked by the QRS complex. If the P wave is morphologically abnormal, atrial (ectopic) tachycardia with abnormal AV nodal conduction is most probable. With specific referral to reentry tachycardia, it is a form of tachycardia in which the conduction from the ventricles back to the atria is by a fast accessory pathway.
permanent junctional reciprocating tachycardia, PJRT	orthodromic AVRT with a slowly conducting accessory pathway, resulting in a long VA incessant tachycardia
heart block	delayed, intermittent, or absent conduction between the atria and the ventricles
first-degree heart block	prolonged PR interval (>0.14 s in fetuses)
second-degree heart block	an occasional non-conducted P wave, resulting in a long RR interval Mobitz I (Wenckebach) type: progressive slowing of the conduction of each subsequent impulse in the AV node, until the node fails to conduct one impulse Mobitz II type: unpredictable failure of the His–Purkinje pathway to conduct some of the impulses from the atria to the ventricles; unlike the Mobitz type I, there is no change in the PR interval prior to the non-conducted P wave
high-degree block	successive non-conducted P waves
complete (third-degree) heart block	also referred to as AV dissociation, absent conduction beteween the atria and the ventricles
